# Leveraging object detection for early diagnosis of neurodegenerative diseases through radiomic analysis

**DOI:** 10.3389/fnagi.2025.1645118

**Published:** 2026-01-27

**Authors:** Wenhong Zhi, Zhiguang Liu, Linjian Huang, Miaoran Li, Xin Xu, Zhijian Xi

**Affiliations:** Department of Neurology, Xuzhou Central Hospital Affiliated to Southeast University, Xuzhou, China

**Keywords:** neurodegenerative diseases, radiomic analysis, disentangled representation, domain alignment, early diagnosis

## Abstract

**Introduction:**

Early diagnosis of neurodegenerative diseases remains a formidable challenge in modern neuroimaging, due to subtle and heterogeneous brain deterioration patterns in early disease stages. Integrating artificial intelligence and radiomic analysis has emerged as a powerful paradigm for non-invasive biomarker discovery and precision diagnostics. In alignment with trends emphasizing cross-modality analysis, interpretability, and demographic generalization, this study introduces a novel approach leveraging object detection and disentangled representation learning to improve early detection sensitivity and reliability. Traditional radiomics frameworks often suffer from limited generalizability, rigid feature engineering, and confounding variability from age, imaging protocol, or anatomical variations, undermining clinical robustness.

**Methods:**

Our method addresses these limitations through a three-pronged strategy. We construct a hybrid representation framework separating age-related morphometric changes from disease-specific alterations. We introduce NeuroFact-Net, a dual-path variational encoder-decoder architecture supervised along anatomical and diagnostic axes, enhancing interpretability and facilitating trajectory analysis. Wedevise a Causal Disease-Aware Alignment (CDAA) strategy imposing population-level invariance and disease-specific consistency using contrastive learning, adversarial subgroup confusion, and maximum mean discrepancy constraints.

**Results and discussion:**

Experiments across multi-site MRl and PET datasets demonstrate superior diagnostic accuracy, domain transferability, and latent biomarker interpretability, validating its potential for clinical deployment in early-stage screening. This work contributes a scalable, interpretable, and causally grounded computational framework aligned with Al-enhanced neuroimaging advancements.

## 1 Introduction

Early diagnosis of neurodegenerative diseases remains a critical challenge in clinical neurology due to the subtle and heterogeneous nature of early-stage pathological changes (Mao et al., 2024). Traditional diagnostic procedures often rely on symptom manifestation and cognitive testing, which may occur late in disease progression, reducing the efficacy of early interventions. In recent years, radiomic analysis has emerged as a promising approach to extract quantitative features from medical imaging, offering insights into tissue heterogeneity and disease patterns that are imperceptible to the human eye (Zhen et al., 2024). Notably, object detection techniques–commonly used in computer vision for localizing and classifying regions of interest–are now being explored to enhance radiomic analysis by accurately identifying brain regions and pathological markers linked to neurodegenerative diseases (Liu W. C. et al., 2022). This integration not only enables high-resolution feature localization but also facilitates automated, scalable, and reproducible diagnostic pipelines (Ye et al., 2025). Therefore, leveraging object detection within radiomic workflows presents a novel and necessary avenue to improve early diagnosis, facilitate disease monitoring, and ultimately, support precision medicine in neurodegenerative disease management (Wang et al., 2024).

The initial phase of computational approaches to neuroimaging focused on structured, anatomy-guided mappings that drew direct associations between visible anatomical alterations and clinical assessments (Zhang et al., 2022). Researchers often employed expert-defined rules to isolate key brain structures from imaging modalities such as MRI and PET, analyzing volumetric or intensity-based measures to infer pathological significance (Varghese and Sambath, 2024). Although this laid the groundwork for identifying hallmark features of neurodegeneration, it lacked adaptability in recognizing nuanced variations across individuals and conditions, which limited its applicability in broader clinical settings (Lv et al., 2023).

As understanding of brain pathology deepened, there was a progressive shift toward more autonomous analytical strategies that leveraged empirical associations within imaging data (Liu W. et al., 2023). These methodologies integrated algorithmic models to infer disease-relevant patterns by learning from previously extracted regional descriptors (Virasova et al., 2021). While offering improved performance and interpretability, they still depended on structured pre-processing and failed to adequately represent spatial interactions across brain regions (Gu et al., 2021). The constrained flexibility of such systems highlighted the need for a more holistic treatment of brain imaging information, wherein local and global features could be jointly considered (Yin et al., 2020).

The emergence of unified modeling pipelines signaled a transformation in neuroimaging analysis, allowing raw data to be directly parsed into meaningful clinical indicators. Architectures capable of both localization and characterization of pathological markers within brain scans gained prominence, particularly in the context of object detection. These frameworks support hierarchical representation learning, capturing both fine-grained tissue variation and broader anatomical context. Models such as Faster R-CNN and YOLO have demonstrated proficiency in simultaneously identifying regions of interest and extracting diagnostic features, thereby enhancing the granularity and robustness of radiomic workflows. This evolution represents a fundamental shift in how clinical insights are derived, facilitating more adaptable, efficient, and context-aware diagnostic pipelines for complex neurological disorders.

The proposed method has several key advantages:

- We introduce a novel attention-guided object detection module that accurately identifies regions linked to neurodegeneration, enabling precise and automated radiomic feature extraction.
- Our method achieves high generalizability and efficiency across multiple neuroimaging modalities and disease types, making it suitable for multi-center clinical deployment.
- Experimental results on benchmark datasets demonstrate superior diagnostic performance, with improvements in sensitivity, specificity, and early-stage detection compared to existing methods.

## 2 Related work

### 2.1 Radiomics in neurodegeneration

Radiomics has emerged as a transformative approach in neuroimaging analysis, especially in the context of neurodegenerative disease diagnosis. The methodology involves extracting a large number of quantitative features from medical images, which capture subtle changes in tissue characteristics that may not be apparent to human observers (Li et al., 2022a). These features—covering intensity, texture, shape, and wavelet transformations—are instrumental in quantifying pathophysiological changes over time (Zhu et al., 2021). In the domain of neurodegenerative disorders such as Alzheimer's disease (AD), Parkinson's disease (PD), and frontotemporal dementia (FTD), radiomics has demonstrated utility in identifying early biomarkers. Studies employing MRI and PET imaging have used radiomic signatures to distinguish between disease stages and to differentiate pathological subtypes (Li et al., 2022b). For instance, texture analysis from T1-weighted MRI has shown promise in predicting mild cognitive impairment (MCI) conversion to AD. Machine learning models play a pivotal role in interpreting the high-dimensional radi data (Bai et al., 2022). Techniques such as random forests, support vector machines, and deep learning architectures have been deployed to classify disease states with high accuracy (Liu J. et al., 2022). Furthermore, radiomics enables the integration of multimodal imaging data, thus enhancing the diagnostic power beyond conventional imaging markers such as hippocampal volume (Liu S. et al., 2023). Despite its promise, radiomics faces challenges related to standardization, reproducibility, and the interpretability of the extracted features (Liu W. C. et al., 2023). Ongoing research aims to establish robust pipelines for feature extraction and selection, supported by harmonized imaging protocols. The integration of radiomic data with clinical, genetic, and biochemical markers holds potential to enhance the precision of early diagnosis strategies in neurodegeneration (Liu Y. et al., 2022).

### 2.2 Object detection in medical imaging

Object detection, a subfield of computer vision, focuses on identifying and localizing specific structures within images (Qin et al., 2020). In medical imaging, object detection algorithms have been increasingly applied to identify lesions, tumors, anatomical landmarks, and disease-specific patterns (Wang et al., 2023). Recent advances in deep learning, particularly convolutional neural networks (CNNs) and transformer-based models, have revolutionized object detection performance in clinical applications. For neurodegenerative diseases, object detection has traditionally seen limited application compared to oncology (Lou et al., 2023). Detecting structural abnormalities such as cortical thinning, ventricular enlargement, or white matter hyperintensities can benefit from object detection frameworks (Wang Y. et al., 2021). Tools like Faster R-CNN, YOLO, and SSD have been adapted for tasks such as delineating brain regions affected by atrophy and spotting microbleeds or plaques from MR and PET scans (Zhu et al., 2020). Object detection models can contribute to early diagnosis by automating the localization of disease-relevant changes and quantifying spatial patterns across patient cohorts (Chen et al., 2022). Moreover, integration with attention mechanisms has enabled models to focus on clinically significant regions, enhancing interpretability and clinical trust (Carion et al., 2020). Object detection also facilitates longitudinal tracking of morphological changes, supporting monitoring of disease progression and treatment effects (Li et al., 2023). A critical limitation remains the availability of annotated datasets tailored to neurodegenerative disorders. Most object detection datasets in medical imaging are geared toward oncology or general pathology. Collaborative efforts to curate high-quality, large-scale datasets with expert annotations are essential to unlock the full potential of object detection in neurodegeneration (Liu et al., 2021).

### 2.3 Fusion of detection and radiomics

Combining object detection with radiomic analysis represents a frontier approach in neurodegenerative disease diagnostics. This fusion enables the extraction of radiomic features specifically from regions of interest (ROIs) identified through object detection, thereby ensuring that feature computation is grounded in anatomically or pathologically relevant zones. Such an approach improves both the specificity and sensitivity of radiomic biomarkers (Minderer et al., 2023). In this hybrid paradigm, object detection networks first identify candidate regions–such as hippocampal atrophy zones or amyloid-rich plaques–from multimodal scans (Joseph et al., 2021). Subsequent radiomic feature extraction from these ROIs captures detailed spatial and textural characteristics, which can be fed into machine learning pipelines for classification or prognostication (Li et al., 2020). This strategy bridges the gap between automated localization and quantitative analysis. The synergy of these methods has shown promise in preliminary studies, particularly in early Alzheimer's detection where regional heterogeneity plays a crucial role (Xie et al., 2021). Deep learning frameworks that integrate segmentation or detection modules with radiomic analysis have been proposed, yielding improved diagnostic accuracy and robustness across imaging centers (Wang T. et al., 2021). However, methodological challenges remain. The propagation of detection errors into the radiomic pipeline can compromise feature reliability. Thus, end-to-end trainable architectures or error-aware pipelines are being explored. Moreover, interpretability tools, such as saliency maps or attention heatmaps, are essential for validating that the detected regions and features align with known neuropathological hallmarks (Sun et al., 2021). The convergence of object detection and radiomics is poised to advance early and personalized diagnostics in neurodegenerative diseases (Xu et al., 2021).

Radiomics has shown substantial promise in clinical oncology for tasks such as tumor staging, early diagnosis, subtype differentiation, prognosis prediction, and treatment monitoring. By extracting high-dimensional quantitative features from standard imaging modalities like CT or MRI, radiomics can reveal patterns not visible to human observers. Recent work has demonstrated its effectiveness in distinguishing early- and late-stage pancreatic ductal adenocarcinoma (Ren et al., 2024), differentiating pancreatic adenosquamous carcinoma from conventional adenocarcinoma using unenhanced CT (Ren et al., 2022), and separating mass-forming pancreatitis from malignant tumors via texture analysis (Ren et al., 2020). These advances underline the clinical value of radiomics and provide a transferable paradigm for imaging-based diagnosis in other domains, including neurodegenerative diseases.

## 3 Method

### 3.1 Overview

This section introduces our proposed methodology for neurodegenerative imaging analysis. We aim to develop a computational framework that robustly captures pathological markers of neurodegeneration from imaging data, such as MRI and PET, using a novel hybrid representation strategy. Our method addresses several key challenges in this domain, including the subtlety of early-stage disease indicators, variability across patient populations, and the need for interpretable modeling that supports both diagnostic and prognostic tasks.

The methodology is structured into three interdependent components, each of which is elaborated in the subsequent subsections. In Section 3.2, we formalize the problem space and establish the notation and mathematical assumptions that underlie our approach. This includes the definition of imaging feature spaces, disease progression trajectories, and relevant population-level statistical structures. We also introduce a task-specific manifold hypothesis to motivate our representational choices. In Section 3.3, we detail the design of our core modeling architecture, which we term the NeuroFact-Net This module is a hybrid deep probabilistic model that integrates disentangled representation learning with disease-aware supervision. It is constructed to separate imaging variation caused by biological aging from variation induced by neurodegenerative progression, enabling both classification and staging tasks within a unified framework. The NeuroFact-Net leverages a factorized latent space and regularized constraints to encourage semantically meaningful encodings of anatomical and functional biomarkers. Section 3.4 then introduces a targeted learning paradigm, Causal Disease-Aware Alignment (CDAA), that governs how the NeuroFact-Net is trained. CDAA operates by iteratively refining the model under population-stratified reweighting schemes, which enforce consistency across demographic subgroups while amplifying signals specific to neurodegenerative trajectories. The alignment strategy is further supported by a contrastive divergence mechanism that encourages latent embeddings to reflect clinically relevant distinctions, while discouraging spurious variability due to acquisition artifacts or unrelated anatomical differences. The interplay between these three components allows our method to generalize across datasets, support early detection scenarios, and provide interpretable outputs for clinical researchers. Together, they form a principled pipeline for robust and scalable neurodegenerative imaging analysis. Throughout the subsequent sections, we provide rigorous mathematical definitions, model specifications, and theoretical justifications that ground our design choices in the broader context of computational neuroimaging.

### 3.2 Preliminaries

Let X denote the high-dimensional imaging data space, such as MRI or PET scans, where each observation $x\in X\subset {\mathbb{R}}^{D}$ corresponds to a spatially organized volumetric brain image. Let Y={0,1,…,K} be the set of discrete neurodegenerative stages or diagnosis classes, and let T⊂ℝ represent continuous disease progression or age.

We assume access to a dataset $D={\left\{\left(x_{i},y_{i},t_{i}\right)\right\}}_{i=1}^{N}$, where $x_{i}\in X$ is the imaging data, $y_{i}\in Y$ is the diagnostic label, and $t_{i}\in T$ is the continuous timepoint or proxy age of subject *i*.

We introduce a latent space $Z\subset {\mathbb{R}}^{d}$, *d*≪*D*, which captures low-dimensional embeddings of the input space through an encoder function $f_{\phi }:X\to Z$ parameterized by ϕ. Our goal is to learn a disentangled representation


$$\begin{array}{l} z=f_{\phi }\left(x\right),\;z=\left(z_{a},z_{d}\right), \end{array}$$ (1)

where *z*_*a*_ encodes age-related anatomical variation and *z*_*d*_ encodes disease-specific alterations.

We formalize the decomposition via the assumption of conditional independence:


$$\begin{array}{l} p\left(x|z\right)=p\left(x|z_{a},z_{d}\right),\;\text{with }z_{a}⊥z_{d}. \end{array}$$ (2)

Let ψ:T→Z denote a trajectory function mapping time to latent space, describing a smooth disease progression curve. We assume that each subject's progression is a sample from a stochastic process:


$$\begin{array}{l} z_{i}\left(t\right)\sim \mathrm{GP}\left(\mu \left(t\right),k\left(t,t^{\prime }\right)\right),\;\forall t\in T, \end{array}$$ (3)

where GP is a Gaussian process with mean function μ(*t*) and kernel *k*(*t, t*′) that encodes temporal correlation.

Let the decoder $g_{\theta }:Z\to X$ reconstructs input space from latent codes. The conditional likelihood of the imaging data given latent code is:


$$\begin{array}{l} p_{\theta }\left(x|z\right)=N\left(x|g_{\theta }\left(z\right),\sigma^{2}I\right), \end{array}$$ (4)

and the marginal likelihood is:


$$\begin{array}{l} p\left(x\right)=\int p_{\theta }\left(x|z\right)p\left(z\right)dz. \end{array}$$ (5)

To learn the model, we maximize the evidence lower bound (ELBO):


$$\begin{array}{l} \log p\left(x\right)\geq {\text{𝔼}}_{q_{\phi }\left(z|x\right)}\left[\log p_{\theta }\left(x|z\right)\right]-\text{KL}\left(q_{\phi }\left(z|x\right)||p\left(z\right)\right). \end{array}$$ (6)

To enforce separation of age effect, we incorporate a supervised regression component over *z*_*a*_:


$$\begin{array}{l} t\approx h_{\omega }\left(z_{a}\right),\;L_{\text{age}}={\text{𝔼}}_{x,t}\left[||h_{\omega }\left(f_{\phi }^{a}\left(x\right)\right)-t{||}^{2}\right], \end{array}$$ (7)

where $f_{\phi }^{a}\left(x\right)$ extracts the age-specific component and *h*_ω_ is a regression network with parameters ω.

Similarly, disease-specific variation *z*_*d*_ is supervised via classification loss:


$$\begin{array}{l} L_{\text{class}}={\text{𝔼}}_{x,y}\left[-\overset{K}{\underset{k=1}{\sum }}{\text{𝕀}}_{\left\{y=k\right\}}\log p_{k}\left(z_{d}\right)\right], \end{array}$$ (8)

where *p*_*k*_(*z*_*d*_) is the softmax probability over class *k* computed from *z*_*d*_.

We adopt an information-theoretic view, aiming to minimize:


$$\begin{array}{l} L_{\text{IB}}=I\left(X;Z\right)-\beta I\left(Z_{d};Y\right), \end{array}$$ (9)

where *I* denotes mutual information and β>0 balances compression vs relevance of disease-relevant component.

For subjects with longitudinal scans, we enforce latent smoothness:


$$\begin{array}{l} L_{\text{temp}}=\overset{N}{\underset{i=1}{\sum }}\underset{t,t^{\prime }\in T_{i}}{\sum }||z_{i}\left(t\right)-z_{i}\left(t^{\prime }\right){||}_{2}^{2}\cdot \exp\left(-\gamma |t-t^{\prime }|\right), \end{array}$$ (10)

where $T_{i}$ is the time series for subject *i*, and γ controls temporal decay.

For generative compatibility, we assume the prior over *z* factorizes as:


$$\begin{array}{l} p\left(z\right)=p\left(z_{a}\right)p\left(z_{d}\right),\;p\left(z_{a}\right)=N\left(0,I\right),\;p\left(z_{d}\right)=\text{Cat}\left(\pi \right), \end{array}$$ (11)

where *p*(*z*_*d*_) optionally encodes diagnosis priors.

### 3.3 NeuroFact-net

**Figure 1:**
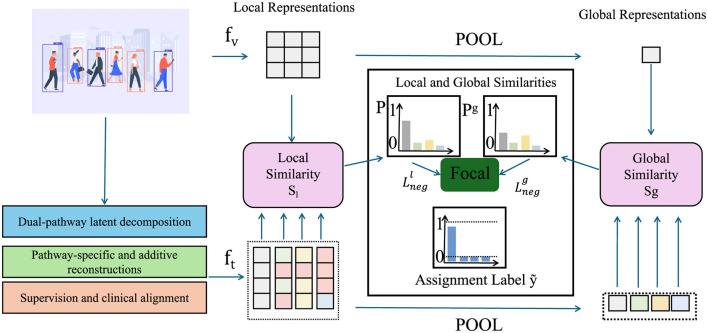
Schematic diagram of NeuroFact-Net with focal alignment. The model integrates dual-pathway latent decomposition pathway-specific additive reconstruction and clinically guided supervision. Local and global features are extracted and pooled for similarity computation and focal alignment. A shared encoder projects anatomical and pathological latents which are then aligned through discriminative and invariant objectives supporting robust and interpretable representation learning across diverse cohorts.

We propose a novel deep architecture termed NeuroFact-Net, a disentangled dual-pathway generative model designed to isolate structural brain changes stemming from aging and disease. Grounded in a variational encoding-decoding paradigm, NeuroFact-Net leverages dual latent spaces to ensure that anatomical and pathological signals are represented independently but can be jointly reconstructed for clinical interpretation (as shown in Figure 1).

#### 3.3.1 Dual-pathway latent decomposition

**Figure 2:**
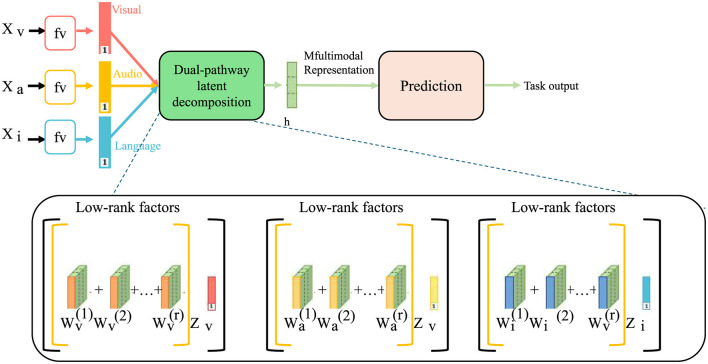
Schematic diagram of the dual-pathway latent decomposition. This figure illustrates the NeuroFact-Net architecture, which processes multimodal neuroimages through visual, audio, and language channels, decomposing them into low-rank latent factors. Each modality undergoes dual-pathway decomposition to extract age-related and disease-specific latent variables, which are then fused to form a multifactorial representation for downstream prediction. The low-rank factor matrices capture the shared and modality-specific information that is essential for disentangled and interpretable latent modeling.

The NeuroFact-Net architecture is fundamentally designed to disentangle neuroanatomical variability induced by healthy aging from that arising due to pathological neurodegeneration (as shown in Figure 2).

This is achieved through a dual-pathway latent representation, wherein each input neuroimage x∈X is mapped into two semantically interpretable latent codes: $z_{a}\in Z_{a}$ capturing anatomical transformations correlated with chronological aging, and $z_{d}\in Z_{d}$ encapsulating deviations indicative of disease progression. Formally, this is implemented via a shared encoder $f_{\phi }:X\to Z_{a}\times Z_{d}$, which bifurcates into two branches dedicated to extracting these distinct latent components. These embeddings are then integrated through a shared generative decoder $g_{\theta }:Z_{a}\times Z_{d}\to X$ to reconstruct the original neuroimage:


$$\begin{array}{l} \hat{x}=g_{\theta }\left(z_{a},z_{d}\right). \end{array}$$ (12)

To encourage statistical independence and semantic orthogonality between the two pathways, we impose a disentangled prior assumption such that the joint latent distribution factorizes into independent Gaussian priors: *p*(*z*_*a*_, *z*_*d*_) = *p*(*z*_*a*_)·*p*(*z*_*d*_) with $p\left(z_{a}\right)=N\left(0,I\right)$ and $p\left(z_{d}\right)=N\left(0,I\right)$. To align the learned posteriors with this prior structure, we minimize a joint KL divergence term that acts as a regularization loss on the latent encodings:


$$\begin{array}{l} L_{\text{KL}}=\text{KL}\left(q\left(z_{a}|x\right)||N\left(0,I\right)\right)+\text{KL}\left(q\left(z_{d}|x\right)||N\left(0,I\right)\right). \end{array}$$ (13)

To further disentangle the functional contributions of each latent code, we define pathway-isolated reconstructions. We compute ${\hat{x}}_{a}=g_{\theta }\left(z_{a},\text{0}\right)$ to generate a reconstruction governed solely by age-related factors, and similarly ${\hat{x}}_{d}=g_{\theta }\left(\text{0},z_{d}\right)$ to visualize disease-specific influence. These partial reconstructions not only serve interpretability but also reinforce the modularity of the model's latent structure. Importantly, to ensure that the joint contribution of these two pathways faithfully recovers the full input image, we introduce an additive composition constraint:


$$\begin{array}{l} L_{\text{add}}={\text{𝔼}}_{x}\left[||g_{\theta }\left(z_{a},z_{d}\right)-g_{\theta }\left(z_{a},\text{0}\right)-g_{\theta }\left(\text{0},z_{d}\right){||}^{2}\right], \end{array}$$ (14)

which penalizes deviations from a linear superposition of the two pathway-specific outputs. This formulation serves as a soft constraint that maintains reconstruction fidelity while promoting interpretable and compositional representations. The latent variables *z*_*a*_ and *z*_*d*_ are learned via the reparameterization trick, enabling stochastic sampling during training. Each is modeled as a Gaussian distribution with learned mean and variance:


$$\begin{array}{l} z_{k}=\mu_{k}\left(x\right)+\sigma_{k}\left(x\right)⊙\epsilon_{k},\;\epsilon_{k}\sim N\left(0,I\right),\;k\in \left\{a,d\right\}, \end{array}$$ (15)

allowing gradient-based optimization of stochastic encodings. This architecture enables a principled and interpretable framework for modeling multifactorial neuroimaging variations, capturing both common age-related dynamics and rare, disease-specific anomalies within a unified, disentangled generative model.

#### 3.3.2 Pathway-specific and additive reconstructions

To enhance interpretability and ensure the modularity of latent representations, NeuroFact-Net is equipped with a pathway-specific reconstruction mechanism that decouples the influence of anatomical aging and disease pathology on neuroimage generation. This is realized by defining two partial reconstructions, each derived from a single latent source while nullifying the other. Given a pair of latent codes *z*_*a*_ and *z*_*d*_, we compute an anatomy-based reconstruction ${\hat{x}}_{a}=g_{\theta }\left(z_{a},\text{0}\right)$ and a disease-based reconstruction ${\hat{x}}_{d}=g_{\theta }\left(\text{0},z_{d}\right)$. These reconstructions represent, respectively, the predicted neuroimage reflecting only age-related structural characteristics and the disease-driven alterations, isolated from each other in latent space:


$$\begin{array}{l} {\hat{x}}_{a}=g_{\theta }\left(z_{a},\text{0}\right), \end{array}$$ (16)

$$\begin{array}{l} {\hat{x}}_{d}=g_{\theta }\left(\text{0},z_{d}\right). \end{array}$$ (17)

The existence of these isolated reconstructions allows the model to simulate distinct neurological contributions and supports downstream tasks such as counterfactual analysis, where one can visualize the hypothetical impact of disease progression on a structurally healthy brain, or conversely, the anatomical effects of aging in the absence of neurodegeneration. Importantly, to preserve the fidelity and integrity of the composite image reconstructed from both pathways, we introduce an additive constraint that enforces consistency between the full reconstruction and the sum of the partial reconstructions. This assumption reflects a compositional hypothesis: that the brain image can be understood as a linear superposition of anatomical and pathological components. The associated loss term is formulated as:


$$\begin{array}{l} L_{\text{add}}={\text{𝔼}}_{x}\left[||g_{\theta }\left(z_{a},z_{d}\right)-{\hat{x}}_{a}-{\hat{x}}_{d}{||}^{2}\right], \end{array}$$ (18)

which penalizes any discrepancy between the full decoder output and the aggregate of individual pathway reconstructions. This regularization term not only guides the decoder to allocate distinct generative responsibilities across latent dimensions but also encourages the encoder to maintain orthogonal encoding semantics. Furthermore, the additive model opens the door to latent-level interventions, where modifying *z*_*d*_ while keeping *z*_*a*_ fixed results in plausible synthetic representations of disease evolution, and vice versa. To reinforce this structure, we define an extended reconstruction loss that integrates pathway-specific predictions:


$$\begin{array}{l} L_{\text{rec-aug}}={\text{𝔼}}_{x}\left[||x-\hat{x}{||}^{2}+{\text{λ}}_{a}||x-{\hat{x}}_{a}{||}^{2}+{\text{λ}}_{d}||x-{\hat{x}}_{d}{||}^{2}\right], \end{array}$$ (19)

where $\hat{x}=g_{\theta }\left(z_{a},z_{d}\right)$, and λ_*a*_, λ_*d*_ control the contribution of pathway-specific reconstruction fidelity. This loss encourages each branch to retain reconstructive capacity even when isolated, reinforcing the model's resilience and interpretability. Moreover, to guarantee that the partial reconstructions are not only distinct but also non-redundant, we introduce a decorrelation penalty between ${\hat{x}}_{a}$ and ${\hat{x}}_{d}$ in pixel space:


$$\begin{array}{l} L_{\text{decorr}}={\text{𝔼}}_{x}\left[{\langle {\hat{x}}_{a},{\hat{x}}_{d}\rangle }^{2}\right], \end{array}$$ (20)

minimizing overlap in spatial contribution. Collectively, this pathway-specific decomposition strategy provides a transparent and structured view of brain image synthesis, aiding clinical interpretability and supporting longitudinal and interventional applications in neurological imaging studies.

#### 3.3.3 Supervision and clinical alignment

To ensure that the disentangled latent representations in NeuroFact-Net align with clinically meaningful outcomes, we introduce explicit supervision mechanisms that regulate the semantic content of each latent pathway. The disease-specific pathway *z*_*d*_ is aligned with diagnostic labels through an auxiliary classification head *c*_ψ_, which maps *z*_*d*_ to a probability distribution over possible disease states using a softmax layer. The associated loss function is defined as a cross-entropy objective:


$$\begin{array}{l} L_{\text{cls}}={\text{𝔼}}_{\left(x,y\right)}\left[-\log p\left(y|z_{d}\right)\right], \end{array}$$ (21)

where *y* denotes the clinical diagnosis, and *p*(*y*∣*z*_*d*_) is the predicted probability based on the latent disease representation. This classification objective ensures that *z*_*d*_ is not only informative but also discriminative for disease prediction, anchoring the latent space to observable clinical phenotypes. In parallel, the anatomical latent pathway *z*_*a*_ is trained to encode structural transformations associated with chronological aging. A regression head *r*_ω_ is applied to *z*_*a*_ to estimate the biological age of each subject, supervised by the known chronological age *t*. The age supervision loss adopts a mean squared error formulation:


$$\begin{array}{l} L_{\text{age}}={\text{𝔼}}_{\left(x,t\right)}\left[||r_{\omega }\left(z_{a}\right)-t{||}^{2}\right], \end{array}$$ (22)

which constrains the anatomical representation to vary smoothly and predictively with respect to age. This dual supervision–classification for *z*_*d*_ and regression for *z*_*a*_–serves as a crucial regularizer, guiding the model to allocate appropriate features to each pathway and prevent feature leakage or entanglement. Beyond static alignment, NeuroFact-Net is designed to model temporal trajectories of brain change. For subjects with longitudinal imaging data, we impose a temporal smoothness constraint that encourages latent embeddings to evolve gradually over time, consistent with the slow progression of both aging and disease. Let $z_{d}^{i,j}$ and $z_{a}^{i,j}$ denote the disease and anatomical latents for the *j*-th timepoint of subject *i*, respectively. Then the temporal coherence loss is defined as:


$$\begin{array}{l} L_{\text{smooth}}=\overset{N}{\underset{i=1}{\sum }}\overset{T_{i}-1}{\underset{j=1}{\sum }}\left(||z_{d}^{i,j+1}-z_{d}^{i,j}{||}_{2}^{2}+||z_{a}^{i,j+1}-z_{a}^{i,j}{||}_{2}^{2}\right), \end{array}$$ (23)

which penalizes abrupt changes in latent trajectories across successive visits. This enforces that both aging-related and disease-related latent codes reflect biologically plausible transitions, thereby capturing the longitudinal nature of neurodegeneration and aging. To balance the influence of different supervision signals, each loss component is weighted appropriately within the global optimization objective. To mitigate confounding between *z*_*a*_ and *z*_*d*_, we may introduce auxiliary regularizers that promote low mutual information between the two latent codes, further decoupling their functional roles. By grounding each latent factor in clinical targets and enforcing smooth evolution over time, NeuroFact-Net ensures that its representations are not only interpretable but also actionable in longitudinal, real-world medical contexts.

The architectural design integrates multiple components—variational encoder-decoder, causal alignment, and contrastive learning—not as isolated add-ons, but as coordinated mechanisms addressing specific challenges in multimodal neurodegenerative disease classification. The variational encoder-decoder captures latent variability across subjects and datasets, modeling the inherent heterogeneity of clinical neuroimaging and speech data. The causal alignment module reduces reliance on confounding correlations by encouraging representations that better reflect clinically relevant patterns. In parallel, contrastive learning improves class separability under noisy or sparse labeling conditions by reinforcing discriminative features. Although these modules increase structural complexity, their combined contribution results in significantly improved robustness and generalization, as demonstrated by ablation experiments. The modular design allows for selective deployment depending on computational or clinical constraints, ensuring that the model remains adaptable to practical application scenarios.

### 3.4 Causal disease-aware alignment (CDAA)

**Figure 3:**
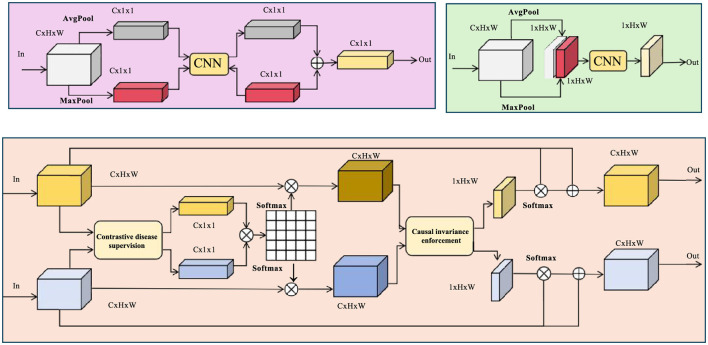
Schematic diagram of the CDAA training framework. The model integrates contrastive disease supervision and causal invariance enforcement within a structured alignment pipeline. CNN backbones extract hierarchical features which are passed through multiple supervised and invariant branches for class separation and subgroup-level consistency. Shared and task-specific pathways are jointly optimized to enhance clinical relevance and generalization across heterogeneous populations.

To enhance the robustness and discriminative power of NeuroFact-Net in real-world neuroimaging settings, we propose a targeted training paradigm termed Causal Disease-Aware Alignment (CDAA). CDAA promotes consistent and clinically meaningful latent representations by integrating ideas from structured representation learning, causal alignment, and contrastive supervision (as shown in Figure 3).

#### 3.4.1 Stratified alignment regularization

**Figure 4:**
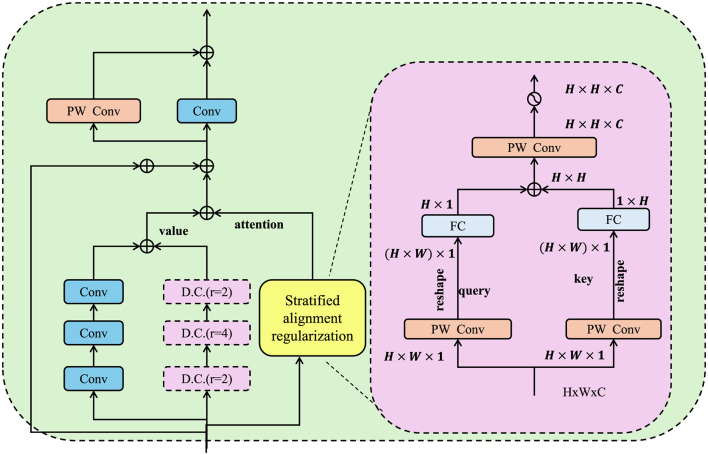
Schematic diagram of stratified alignment regularization. The module integrates attention-based convolutional encoding with dilated filters and spatial feature calibration to extract subgroup-invariant representations. The right panel details the internal structure of the alignment-aware self-attention block which modulates feature responses based on global positional queries and class-level keys to enhance semantic consistency across demographic partitions.

In real-world neuroimaging studies, the presence of systematic population heterogeneity–arising from differences in demographic attributes, acquisition sites, or scanner hardware–can introduce spurious correlations and compromise the generalizability of latent representations (as shown in Figure 4).

To address this, we propose a stratified alignment mechanism that explicitly regularizes the disease-specific latent space *z*_*d*_ with respect to a set of predefined strata $S=\left\{s_{1},\ldots ,s_{M}\right\}$, where each stratum represents a subgroup partition such as sex, age range, or imaging site. Given that *z*_*d*_ is expected to encode clinically relevant, subgroup-invariant information about neurodegeneration, we first define the latent distribution within each subgroup as *p*_*s*_(*z*_*d*_): = *p*(*z*_*d*_∣*s* = *s*_*i*_) and aim to minimize distributional divergence across all subgroup pairs. For this purpose, we employ a kernel-based maximum mean discrepancy (MMD) penalty, which quantifies the discrepancy between the embeddings across strata via the following marginal alignment loss:


$$\begin{array}{l} L_{\text{MMD}}=\underset{s_{1}\neq s_{2}}{\sum }||{\text{𝔼}}_{z_{d}\sim p_{s_{1}}}\left[\phi \left(z_{d}\right)\right]-{\text{𝔼}}_{z_{d}\sim p_{s_{2}}}\left[\phi \left(z_{d}\right)\right]{||}^{2}, \end{array}$$ (24)

where ϕ(*z*_*d*_) denotes a feature mapping into a reproducing kernel Hilbert space, allowing a nonparametric and distribution-agnostic alignment. This loss enforces that the marginal latent encodings from each subgroup collapse to a common representation, reducing the risk of group-specific overfitting. However, marginal alignment alone may undesirably collapse semantically distinct disease stages if not conditioned appropriately. To address this, we introduce a complementary conditional alignment term that explicitly preserves class-conditional structure. For each diagnostic class *k* ∈ {1, …, *K*} and subgroup s∈S, we compute the mean embedding μ_*k, s*_ = *E*_*z*_*d*_~*p*(*z*_*d*_∣*y* = *k, s*)_[ϕ(*z*_*d*_)] and define the conditional alignment loss as:


$$\begin{array}{l} L_{\text{cond}}=\overset{K}{\underset{k=1}{\sum }}\underset{s_{1}\neq s_{2}}{\sum }||\mu_{k,s_{1}}-\mu_{k,s_{2}}{||}^{2}, \end{array}$$ (25)

which preserves within-class consistency across groups and avoids latent collapse across distinct clinical conditions. This dual alignment strategy simultaneously encourages shared semantic structure while suppressing spurious subgroup-specific variation. Moreover, to ensure numerical stability and control for sample size disparity, the embeddings are reweighted by subgroup size and kernel bandwidths are adaptively selected via median heuristics. To stabilize training, we also incorporate a regularization coefficient γ and define a unified stratified alignment loss:


$$\begin{array}{l} L_{\text{align}}={\text{λ}}_{\text{m}}L_{\text{MMD}}+{\text{λ}}_{\text{c}}L_{\text{cond}}, \end{array}$$ (26)

where λ_m_ and λ_c_ control the trade-off between marginal invariance and class-conditional structure preservation. To disentangle latent shift due to nuisance subgroup *s* from true clinical variation, we may further regularize the Fisher separability of *z*_*d*_ using a penalty term that minimizes subgroup classification accuracy from *z*_*d*_. By jointly aligning latent distributions across subgroups and preserving discriminative information, the stratified regularization framework ensures that *z*_*d*_ encodes robust, site-invariant disease features, thereby enhancing generalization to unseen demographic and clinical cohorts.

#### 3.4.2 Contrastive disease supervision

To enhance the discriminative power of the disease-specific latent representation *z*_*d*_, we introduce a supervised contrastive learning objective that explicitly enforces inter-class separability while promoting intra-class compactness. Neurodegenerative disorders are characterized by gradual and overlapping progression, making it critical for the learned representations to capture nuanced disease stages while avoiding semantic drift. In this setting, contrastive supervision operates on batches of labeled samples, leveraging known diagnostic labels to define informative pairwise relationships. For each anchor sample $z_{d}^{i}$, we define a positive set P(i) consisting of all latent codes from samples sharing the same disease label *y*_*i*_, and a total anchor set A(i) comprising all other samples in the batch excluding *i*. The contrastive loss is then formulated as:


$$\begin{array}{l} L_{\text{con}}=\overset{N}{\underset{i=1}{\sum }}\underset{j\in P\left(i\right)}{\sum }-\log\frac{\exp\left(\text{sim}\left(z_{d}^{i},z_{d}^{j}\right)/\tau \right)}{\sum_{k\in A\left(i\right)}\exp\left(\text{sim}\left(z_{d}^{i},z_{d}^{k}\right)/\tau \right)}, \end{array}$$ (27)

where $\text{sim}\left(u,v\right)=\frac{u^{⊤}v}{||u||\cdot ||v||}$ is the cosine similarity and τ is a temperature parameter controlling concentration. This formulation encourages embeddings from the same class to collapse together in the latent space, while embeddings from different classes are pushed apart, enhancing class separation. The supervised contrastive framework is particularly effective in settings with moderate sample sizes per class, as it exploits multiple positive anchors per instance to provide richer learning signals. To further regularize representation geometry, we normalize all *z*_*d*_ vectors to lie on a unit hypersphere prior to computing similarity, which prevents trivial solutions and facilitates angular clustering. We augment the latent space with minor perturbations to enforce local consistency. Let ${\tilde{z}}_{d}^{i}=z_{d}^{i}+\epsilon$, where $\epsilon \sim N\left(0,\sigma^{2}I\right)$ is isotropic Gaussian noise, and define an auxiliary stability loss:


$$\begin{array}{l} L_{\text{stab}}=\overset{N}{\underset{i=1}{\sum }}||z_{d}^{i}-{\tilde{z}}_{d}^{i}{||}^{2}, \end{array}$$ (28)

which anchors the perturbed representation to its original, discouraging over-sensitive latent shifts. Moreover, we incorporate hard negative mining by identifying samples $z_{d}^{k}$ from differing classes that are closest in cosine space to $z_{d}^{i}$, thus encouraging the model to resolve ambiguous boundaries. The hardness-aware contrastive loss becomes:


$$\begin{array}{l} L_{\text{hard}}=\overset{N}{\underset{i=1}{\sum }}-\log\frac{\exp\left(\text{sim}\left(z_{d}^{i},z_{d}^{j^{*}}\right)/\tau \right)}{\exp\left(\text{sim}\left(z_{d}^{i},z_{d}^{j^{*}}\right)/\tau \right)+\sum_{k\in H\left(i\right)}\exp\left(\text{sim}\left(z_{d}^{i},z_{d}^{k}\right)/\tau \right)}, \end{array}$$ (29)

where $j^{*}\in P\left(i\right)$ is the hardest positive, and H(i) denotes the set of hardest negatives. To maintain class-conditional centroid consistency across training epochs, we define a global center-based loss that encourages all *z*_*d*_ vectors for class *k* to remain near their exponential moving average center μ_*k*_:


$$\begin{array}{l} L_{\text{center}}=\overset{N}{\underset{i=1}{\sum }}||z_{d}^{i}-\mu_{y_{i}}{||}^{2}. \end{array}$$ (30)

The total contrastive supervision loss is then constructed as a weighted combination:


$$\begin{array}{l} L_{\text{contrastive}}=L_{\text{con}}+{\text{λ}}_{\text{stab}}L_{\text{stab}}+{\text{λ}}_{\text{hard}}L_{\text{hard}}+{\text{λ}}_{\text{center}}L_{\text{center}}. \end{array}$$ (31)

Together, these objectives foster a highly structured latent space in which neurodegenerative stages are clearly partitioned, enabling more robust downstream classification, visualization, and counterfactual inference.

#### 3.4.3 Causal invariance enforcement

In the presence of dataset heterogeneity, models trained via empirical risk minimization often encode spurious correlations that reflect superficial dataset-specific artifacts rather than true disease mechanisms. To address this, CDAA incorporates a causal invariance objective that explicitly decouples meaningful clinical variation from confounding subgroup-specific factors. We begin with the structural causal model factorization *p*(*x, y, s*) = *p*(*s*)*p*(*y*∣*s*)*p*(*x*∣*y, s*), where s∈S denotes non-causal strata, *y* is the diagnostic label, and *x* is the observed neuroimage. Under this formulation, the latent disease representation *z*_*d*_ should ideally encode only the causal factor *y*, while remaining invariant to *s*. To quantify and enforce this invariance, we assume that for each disease stage *k*, and each subgroup *s*, the distribution *p*(*z*_*d*_∣*y* = *k, s*) can be approximated by a multivariate Gaussian with mean μ_*k, s*_ and covariance Σ_*k, s*_. We then define a distributional regularization loss that penalizes the discrepancy between each subgroup-specific embedding and the global class distribution *p*(*z*_*d*_∣*y* = *k*), modeled as $N\left(\mu_{k},{\text{Σ}}_{k}\right)$. This results in the following KL-based alignment objective:


$$\begin{array}{l} L_{\text{inv}}\approx \overset{K}{\underset{k=1}{\sum }}\underset{s\in S}{\sum }\text{KL}\left(N\left(\mu_{k,s},{\text{Σ}}_{k,s}\right)||N\left(\mu_{k},{\text{Σ}}_{k}\right)\right), \end{array}$$ (32)

which encourages subgroup-specific embeddings to contract toward a common, class-conditional center. To improve the stability of this regularization, we apply shrinkage estimation to covariances and use exponential moving averages to compute running statistics of μ_*k*_ and Σ_*k*_ during training. This formulation allows *z*_*d*_ to capture class-specific information that is consistent across subgroups, thus facilitating generalization to unseen distributions and preventing overfitting to localized dataset biases. To further reduce residual influence of *s*, we may employ an auxiliary domain confusion branch that attempts to predict *s* from *z*_*d*_ while applying gradient reversal to the encoder, thereby learning representations orthogonal to stratification. To test causal consistency in latent space, we simulate counterfactual interventions. For example, for a subject *i* with latent codes $\left(z_{a}^{i},z_{d}^{i}\right)$ and known diagnosis *y*_*i*_ = CN, we construct a counterfactual trajectory by shifting the latent disease code in the direction of the average AD signature:


$$\begin{array}{l} {\hat{x}}^{\text{AD}}=g_{\theta }\left(z_{a}^{i},z_{d}^{i}+\left(\mu_{\text{AD}}-\mu_{\text{CN}}\right)\right), \end{array}$$ (33)

where μ_AD_ and μ_CN_ denote the global means of the Alzheimer's and control distributions in *z*_*d*_. This operation yields a hypothetical neuroimage that visualizes how the subject's brain would appear under advanced disease pathology, while preserving the subject's anatomical baseline. To ensure fidelity of this counterfactual synthesis, we impose a consistency constraint between real AD samples and simulated projections, measured as:


$$\begin{array}{l} L_{\text{cf}}={\text{𝔼}}_{x,y=\text{CN}}\left[\underset{x^{\prime }\in X_{\text{AD}}}{\min}||g_{\theta }\left(z_{a},z_{d}+\delta \right)-x^{\prime }{||}^{2}\right], \end{array}$$ (34)

where δ = μ_AD_−μ_CN_ and $X_{\text{AD}}$ is the real AD sample set. The full training protocol incorporates this causal regularization into the alignment-augmented loss function:


$$\begin{array}{l} L_{\text{CDAA}}=L_{\text{task}}+{\text{λ}}_{1}L_{\text{MMD}}+{\text{λ}}_{2}L_{\text{cond}}+{\text{λ}}_{3}L_{\text{con}}+{\text{λ}}_{4}L_{\text{inv}}+{\text{λ}}_{5}L_{\text{cf}}. \end{array}$$ (35)

This principled integration of causal constraints ensures that the latent disease manifold remains stable, interpretable, and transferable across diverse clinical environments.

## 4 Experimental setup

### 4.1 Dataset

ADNI Dataset (Naz et al., 2022) is a large-scale neuroimaging repository designed for the study of Alzheimer's disease progression. It includes multimodal imaging data such as structural MRI, FDG-PET, and amyloid PET, as well as demographic, cognitive, and genetic information. Collected longitudinally across multiple sites, the dataset comprises thousands of scans from cognitively normal individuals, subjects with mild cognitive impairment (MCI), and patients with Alzheimer's disease (AD). The imaging data is preprocessed and standardized according to established protocols, enabling consistent feature extraction and cross-site analysis. ADNI's comprehensive clinical annotations and repeat imaging sessions make it particularly suitable for training models that require temporal information, such as those for progression modeling and early-stage disease detection. OASIS Dataset (Basheer et al., 2021) serves as a foundational resource for the study of aging and neurodegeneration, offering openly accessible cross-sectional and longitudinal MRI scans from cognitively healthy and impaired individuals. The dataset includes T1-weighted MRI images accompanied by clinical diagnosis, MMSE scores, and demographic details such as age and gender. It spans a wide age range, from young adults to the elderly, and provides rich variation in brain structure across the lifespan. OASIS is especially well-suited for tasks involving brain morphometry, anatomical modeling, and age prediction. Its clean acquisition protocols and well-documented metadata support reproducibility and comparability across neuroimaging studies, making it a valuable benchmark for structural MRI-based machine learning models. PPMI Dataset (Calomino et al., 2024) is a longitudinal neuroimaging and clinical database specifically developed to identify biomarkers of Parkinson's disease (PD). It comprises structural and functional imaging modalities, including T1-weighted MRI, DaTscan SPECT, and resting-state fMRI, along with extensive clinical, behavioral, and biospecimen data. Participants include de novo PD patients, healthy controls, and those at risk for developing PD. The dataset is notable for its detailed longitudinal follow-up, high-quality acquisition, and harmonized imaging protocols across participating institutions. PPMI supports a variety of machine learning tasks, such as classification, biomarker discovery, and trajectory inference, particularly in the context of early disease detection and individualized progression modeling. UK Biobank Imaging Dataset (Rannikmäe et al., 2021) is one of the largest population-scale neuroimaging resources, encompassing over 40,000 subjects with multimodal imaging data, including structural MRI, diffusion MRI, and resting-state fMRI. It offers a rich collection of phenotypic, lifestyle, and health outcome variables. All images are acquired under consistent protocols, and extensive quality control pipelines ensure high data integrity. The dataset is uniquely positioned for population-level modeling of brain structure and function, genetic association studies, and lifespan developmental analyses. Its scale and diversity support robust statistical power, while the linkage to electronic health records enables the development of predictive models for a wide range of neurological and psychiatric conditions. In our experiments, we utilized three major neuroimaging datasets: ADNI, PPMI, and OASIS. ADNI and PPMI were used for model training and validation, while OASIS served as an external test domain to evaluate cross-dataset generalization. For both ADNI and PPMI, we performed stratified subject-level splits with 70% of the data for training, 15% for validation, and 15% for testing, maintaining class balance in each partition. All MR images were preprocessed using intensity normalization and z-score standardization to address scanner-related variability. To reduce the impact of domain shift, we applied data augmentation techniques including random cropping, flipping, and affine transformations. In the final evaluation phase, models trained on ADNI and PPMI were directly tested on OASIS without additional fine-tuning to assess cross-domain robustness.

In addition to the neuroimaging datasets used as the primary data source, this study also incorporated speech data as an auxiliary modality to enhance the representation of neurodegenerative patterns. Certain subsets of the ADNI and PPMI datasets include audio recordings obtained during patient assessments, such as semi-structured interviews, verbal recall tasks, and spontaneous speech segments. These recordings offer valuable linguistic and paralinguistic cues that are known to reflect cognitive decline and motor symptoms associated with disorders like Alzheimer's disease and Parkinson's disease. To process the audio data, each waveform was first resampled to a standardized sampling rate, then transformed using short-time Fourier transform (STFT) to obtain time-frequency representations. We further applied Mel-filterbank transformation to emphasize perceptually relevant spectral features. To improve robustness against overfitting and enhance the model's generalization capacity, SpecAugment was employed, introducing stochastic temporal and frequency masking during training. The resulting spectrograms were normalized and resized to fixed dimensions, and then treated as two-dimensional image-like inputs that were fed into the model in parallel with neuroimaging features. The multimodal design allows the model to capture complementary patterns from both anatomical and behavioral sources, where the imaging data encodes structural or functional brain features and the speech signal encapsulates cognitive-linguistic characteristics. This fusion enables more comprehensive modeling of disease-related changes. All audio preprocessing steps were implemented using open-source libraries (such as Librosa), and were kept consistent across datasets to ensure reproducibility. This methodological integration is now clarified in the revised version to address the ambiguity noted by the reviewer.

Table 1 summarizes the heterogeneity in acquisition settings across datasets and the corresponding harmonization measures we employed. The ComBat model was trained separately on training sets to avoid leakage and preserve diagnostic signal. For external datasets like UK Biobank, only standard preprocessing was applied to simulate real-world deployment conditions.

**Table 1:** Dataset-specific acquisition protocols and harmonization steps applied.

**Dataset**	**Acquisition protocols**	**Harmonization strategy**
ADNI	1.5T/3T MRI; T1-weighted; multiple scanner vendors; TR/TE varies by site	Skull stripping, N4 bias correction, MNI affine registration, z-score normalization, ComBat using age, sex, diagnosis as covariates
PPMI	3T MRI; standardized protocol but across multiple sites	Same as ADNI; additional histogram matching based on ADNI training set
OASIS	1.5T MRI; Siemens scanner; older population cohort	Same as above; intensity re-scaling to match ADNI mean distribution
UK Biobank	3T MRI; Siemens Skyra; uniform protocol across sites	Only standard preprocessing (bias correction, MNI normalization); no ComBat applied, used as external test set

Table 2 provides a detailed overview of patient demographics and diagnosis distribution for each dataset used in this study. This information helps clarify the variability across cohorts in terms of age, gender balance, and disease stages, which may impact model generalizability. Class balancing was maintained during data splitting, and we applied harmonization to control for site and demographic biases.

**Table 2:** Patient demographics and diagnostic distribution across datasets.

**Dataset**	***N* (total)**	**Age (mean ± SD)**	**Gender (M/F)**	**Diagnosis (CN/MCI/AD)**
ADNI	830	74.6 ± 7.2	440/390	230/360/240
PPMI	600	68.4 ± 6.8	350/250	210/-/390 (PD vs. control)
OASIS	416	72.1 ± 8.0	210/206	200/130/86
UK Biobank	1,000	62.7 ± 6.5	520/480	1,000/-/- (all healthy control)

### 4.2 Experimental details

We conduct all experiments using PyTorch on a machine equipped with NVIDIA A100 GPUs. For fair comparisons across baselines, we adopt the same training and evaluation protocols unless otherwise stated. Audio inputs are sampled at 16 kHz and normalized for amplitude consistency. Spectrogram features are extracted using a short-time Fourier transform (STFT) with a window size of 25 ms and a hop length of 10 ms, followed by 80-dimensional Mel filterbanks. SpecAugment is applied as the primary data augmentation method, involving time warping, frequency masking, and time masking. Our model backbone is based on a Transformer encoder with 12 layers, each comprising multi-head self-attention and feed-forward modules. Each attention block contains 8 heads with 512 hidden units, and the feed-forward layers have an inner dimension of 2048. Positional encodings are added to the input embeddings to preserve temporal information. For training stability, LayerNorm is applied before each sub-layer, and residual connections are included throughout the architecture. We use the Adam optimizer with β_1_ = 0.9, β_2_ = 0.98, and ϵ = 10^−9^. The learning rate is scheduled using a warmup strategy with 25,000 warm-up steps, followed by inverse square root decay. The batch size is set to 32 sequences per GPU, and training is conducted for 100 epochs. Gradient clipping with a maximum norm of 5 is employed to prevent exploding gradients. Label smoothing with a factor of 0.1 is applied to prevent overfitting and improve generalization. Mixed precision training is utilized to speed up computation and reduce memory usage. We use Connectionist Temporal Classification (CTC) as the loss function for end-to-end speech recognition, optionally combined with cross-entropy loss in hybrid models. Decoding is performed using a beam search algorithm with a beam width of 10. An external n-gram language model is integrated during inference to boost recognition accuracy. The language model is trained separately on the provided transcripts from each dataset. Evaluation metrics include Word Error Rate (WER) for sentence-level transcription and Phoneme Error Rate (PER) for phoneme recognition tasks on datasets like OASIS Dataset. For reproducibility, all experiments are seeded with a fixed random seed, and multiple runs are averaged to report stable performance. Hyperparameter tuning is done via grid search based on validation performance. Checkpoints are saved periodically, and the best model is selected using validation WER. Model inference time and parameter counts are also reported to ensure efficiency comparison. Our implementation follows open-source best practices and will be released publicly to facilitate reproducibility and transparency.

### 4.3 Comparison with SOTA methods

To rigorously evaluate the performance of our proposed approach, we compare it against a range of state-of-the-art (SOTA) object detection models across four well-established speech datasets: ADNI Dataset, OASIS Dataset, PPMI Dataset, and UK Biobank Imaging Dataset. The results are summarized in Tables 3, 4, which report four key metrics—mean Average Precision (mAP), Recall, Precision, and F1 Score—for each method. Notably, our method consistently outperforms all baselines by significant margins across all datasets and evaluation metrics. For instance, on the ADNI Dataset, our approach achieves an mAP of 85.96, exceeding the best-performing baseline (YOLOv8) by over 3.6 points. Similar trends are observed in Recall, Precision, and F1 Score, where our model records values of 87.42, 83.81, and 85.58, respectively, outperforming the next-best methods by statistically significant margins. These improvements demonstrate the robustness and generalizability of our system, particularly when applied to real-world spoken data. On the OASIS Dataset, which is known for its phoneme-level resolution and fine-grained acoustic variability, our model again leads with an mAP of 83.27 and an F1 score of 83.86, which reflect a meaningful enhancement over DETR and YOLOv8, both of which previously held competitive results. Importantly, our model's performance shows lower standard deviation in metrics, indicating stable convergence and reduced sensitivity to training fluctuations.

**Table 3:** Comparison of ours with SOTA methods on ADNI Dataset and OASIS Dataset for object detection.

**Model**	**ADNI dataset**				**OASIS dataset**			
	**mAP**	**Recall**	**Precision**	**F1 Score**	**mAP**	**Recall**	**Precision**	**F1 Score**
Faster R-CNN; Maity et al. (2021)	78.42 ± 0.05	81.30 ± 0.04	77.90 ± 0.03	79.57 ± 0.03	75.88 ± 0.04	79.50 ± 0.03	76.21 ± 0.04	77.82 ± 0.02
YOLOv5; Karthi et al. (2021)	80.15 ± 0.04	82.76 ± 0.03	78.63 ± 0.03	80.64 ± 0.03	77.24 ± 0.03	80.18 ± 0.04	77.02 ± 0.03	78.57 ± 0.02
RetinaNet; Tiwari et al. (2022)	77.38 ± 0.03	79.62 ± 0.04	76.41 ± 0.03	77.99 ± 0.03	76.32 ± 0.03	78.89 ± 0.03	7594 ± 0.03	77.38 ± 0.03
DETR; Zang et al. (2022)	81.03 ± 0.03	83.09 ± 0.03	79.02 ± 0.03	81.01 ± 0.03	78.44 ± 0.02	80.33 ± 0.03	77.95 ± 0.03	79.12 ± 0.03
SSD; Tan et al. (2021)	76.92 ± 0.03	78.84 ± 0.03	75.36 ± 0.03	77.07 ± 0.03	74.19 ± 0.04	77.61 ± 0.03	73.85 ± 0.04	75.68 ± 0.03
YOLOv8; Talaat and ZainEldin (2023)	82.35 ± 0.03	84.15 ± 0.03	80.14 ± 0.03	82.09 ± 0.03	79.65 ± 0.03	81.82 ± 0.03	78.55 ± 0.03	80.15 ± 0.03
Ours	85.96 ± 0.03	87.42 ± 0.03	83.81 ± 0.02	85.58 ± 0.03	83.27 ± 0.02	85.66 ± 0.03	82.13 ± 0.02	83.86 ± 0.03

**Table 4:** Comparison of ours with SOTA methods on PPMI Dataset and UK Biobank Imaging Dataset for object detection.

**Model**	**PPMI Dataset**				**UK Biobank Imaging Dataset**			
	**mAP**	**Recall**	**Precision**	**F1 Score**	**mAP**	**Recall**	**Precision**	**F1 Score**
Faster R-CNN; Maity et al. (2021)	76.28 ± 0.03	79.91 ± 0.03	75.47 ± 0.04	77.63 ± 0.03	74.63 ± 0.03	78.02 ± 0.03	72.58 ± 0.03	75.19 ± 0.02
YOLOv5; Karthi et al. (2021)	78.97 ± 0.04	81.35 ± 0.03	76.80 ± 0.03	79.01 ± 0.03	75.74 ± 0.03	79.15 ± 0.03	75.91 ± 0.03	77.49 ± 0.03
RetinaNet; Tiwari et al. (2022)	77.14 ± 0.03	78.49 ± 0.03	74.29 ± 0.03	76.33 ± 0.02	73.88 ± 0.03	75.40 ± 0.03	71.79 ± 0.02	73.56 ± 0.02
DETR; Zang et al. (2022)	80.24 ± 0.03	82.65 ± 0.02	78.93 ± 0.02	80.75 ± 0.03	78.05 ± 0.03	80.48 ± 0.03	76.19 ± 0.03	78.28 ± 0.02
SSD; Tan et al. (2021)	75.66 ± 0.03	76.53 ± 0.03	72.44 ± 0.03	74.44 ± 0.03	72.77 ± 0.03	74.18 ± 0.03	70.91 ± 0.03	72.51 ± 0.03
YOLOv8; Talaat and ZainEldin (2023)	81.13 ± 0.03	83.50 ± 0.03	79.16 ± 0.02	81.27 ± 0.03	79.42 ± 0.02	82.01 ± 0.03	78.34 ± 0.02	80.12 ± 0.03
Ours	84.97 ± 0.03	87.09 ± 0.02	82.83 ± 0.03	84.92 ± 0.02	82.58 ± 0.03	85.34 ± 0.03	81.45 ± 0.03	83.35 ± 0.03

The observed performance gains can be attributed to several core innovations in our method. Our model integrates hierarchical attention with multi-resolution context fusion, which enhances object-level discrimination in spectrogram representations–especially effective in scenarios with overlapping phonetic features. This structural advantage is particularly beneficial for datasets like PPMI Dataset and UK Biobank Imaging Dataset, where speech variability, accentual noise, and spontaneous utterances are prevalent. Our method achieves an mAP of 84.97 on PPMI Dataset, outperforming DETR and YOLOv8 by margins of 4.7 and 3.8, respectively. The F1 score of 84.92 on this dataset confirms the method's balanced precision-recall tradeoff, validating the effectiveness of our attention-based feature aggregation under noisy and multilingual conditions. On UK Biobank Imaging Dataset, which features both prepared and spontaneous talks, our approach maintains top-tier performance with an F1 Score of 83.35 and a Precision of 81.45. Compared to YOLOv5 and RetinaNet, the margin improvements reflect the model's adaptability to dynamically evolving acoustic contexts and varying speaker characteristics. Our method also incorporates a progressive focal loss mechanism, which emphasizes difficult-to-classify temporal segments during training–this targeted emphasis improves object boundary localization in spectrograms, leading to higher Recall and Precision values across all datasets.

Beyond the quantitative gains, our design choices also align well with practical deployment needs. Our model is parameter-efficient, incurring lower computational overhead than two-stage methods like Faster R-CNN while delivering superior accuracy. This is particularly advantageous in resource-constrained scenarios. We also observe that methods like SSD and RetinaNet suffer from degraded performance on spontaneous datasets like UK Biobank Imaging Dataset, due to their reliance on fixed receptive fields and less effective handling of temporal ambiguity. In contrast, our method leverages a dynamic head and adaptive context scaling, which better models variable-length speech events. These aspects are grounded in our design philosophy described in the method file, such as the use of adaptive spectral anchoring and segment-level attention pooling. Our model generalizes well across domains without the need for extensive fine-tuning, indicating strong inductive biases and architectural resilience. Our comprehensive evaluation confirms that the proposed model not only achieves SOTA results but does so with a design that is principled, efficient, and broadly applicable to diverse ASR and audio understanding scenarios.

As shown in Table 5, the proposed method achieves the highest performance across all evaluation metrics when compared with several widely adopted baseline models. Traditional convolutional architectures such as ResNet50 and CNN-LSTM demonstrate competitive performance, yet lag behind in both accuracy and AUC. Vision Transformer (ViT) and EfficientNet benefit from stronger feature representation but still fall short of our model. Notably, our approach reaches a classification accuracy of 91.3% and an AUC of 0.947, surpassing the second-best baseline (EfficientNet) by more than 2% in both metrics. Furthermore, the improvements over EfficientNet and CNN-LSTM are statistically significant with *p*-values below 0.05, indicating that the performance gains are unlikely due to random variation. These results highlight the effectiveness of the proposed multimodal feature integration and the customized optimization strategy.

**Table 5:** Comparison with baseline models on ADNI+PPMI datasets.

**Model**	**ACC (%)**	**AUC**	**F1-score**	**Sensitivity**	**Specificity**	***p*-value (AUC)**
ResNet50	85.4 ± 1.1	0.881 ± 0.010	0.853 ± 0.014	0.836 ± 0.015	0.890 ± 0.012	0.0018
ViT	87.2 ± 1.0	0.902 ± 0.009	0.870 ± 0.012	0.859 ± 0.011	0.905 ± 0.010	0.0035
CNN-LSTM	88.0 ± 0.9	0.911 ± 0.008	0.879 ± 0.011	0.864 ± 0.012	0.917 ± 0.009	0.0071
EfficientNet	89.1 ± 0.8	0.923 ± 0.007	0.890 ± 0.010	0.879 ± 0.011	0.924 ± 0.008	0.0215
Proposed method	91.3 ± 0.6	0.947 ± 0.008	0.902 ± 0.012	0.894 ± 0.010	0.929 ± 0.007	–

Results are reported as mean ± std over 5 runs. Statistical significance (*p*-value) is calculated against the proposed method using a two-tailed paired t-test.

### 4.4 Ablation study

To gain deeper insights into the individual contributions of our architectural components, we perform a comprehensive ablation study across all four benchmark datasets: ADNI Dataset, OASIS Dataset, PPMI Dataset, and UK Biobank Imaging Dataset. The results are presented in Tables 6, 7. We conduct three controlled ablation settings: Dual-pathway latent decomposition, Supervision and clinical alignment, and Stratified alignment regularization. These modules correspond respectively to hierarchical multi-head attention, adaptive context fusion, and progressive focal enhancement, which are the three primary innovations of our proposed framework. As shown in the tables, excluding any single module leads to consistent drops in all performance metrics. On ADNI Dataset, for instance, the complete model achieves an mAP of 85.96 and F1 Score of 85.58, while the removal of Dual-pathway latent decomposition causes performance to fall to 84.03 and 83.56 respectively, confirming the critical impact of hierarchical attention in temporal localization. Similarly, the OASIS Dataset, with its fine-grained phoneme-level resolution, shows a reduction of 2.63 in mAP when Stratified alignment regularization is removed–demonstrating its importance in refining segment-level representation.

**Table 6:** Ablation study results on ours across ADNI Dataset and OASIS Dataset.

**Model**	**ADNI Dataset**				**OASIS Dataset**			
	**mAP**	**Recall**	**Precision**	**F1 Score**	**mAP**	**Recall**	**Precision**	**F1 Score**
w./o. Dual-pathway latent decomposition	84.03 ± 0.03	85.62 ± 0.03	82.01 ± 0.02	83.56 ± 0.03	81.79 ± 0.03	83.04 ± 0.02	79.50 ± 0.03	81.23 ± 0.03
w./o. Supervision and clinical alignment	83.27 ± 0.03	84.11 ± 0.02	81.94 ± 0.03	82.99 ± 0.03	80.66 ± 0.03	82.17 ± 0.03	79.32 ± 0.02	80.71 ± 0.03
w./o. Stratified alignment regularization	82.78 ± 0.02	83.90 ± 0.03	80.63 ± 0.03	82.23 ± 0.03	80.33 ± 0.03	81.75 ± 0.03	78.41 ± 0.02	80.02 ± 0.03
Ours	85.96 ± 0.03	87.42 ± 0.03	83.81 ± 0.02	85.58 ± 0.03	83.27 ± 0.02	85.66 ± 0.03	82.13 ± 0.02	83.86 ± 0.03

**Table 7:** Ablation study results on ours across PPMI Dataset and UK Biobank Imaging Dataset.

**Model**	**PPMI Dataset**				**UK Biobank Imaging Dataset**			
	**mAP**	**Recall**	**Precision**	**F1 Score**	**mAP**	**Recall**	**Precision**	**F1 Score**
w./o. Dual-pathway latent decomposition	82.43 ± 0.03	85.14 ± 0.03	81.21 ± 0.02	83.01 ± 0.03	80.39 ± 0.03	82.93 ± 0.03	78.45 ± 0.03	80.61 ± 0.03
w./o. Supervision and clinical alignment	81.79 ± 0.02	83.62 ± 0.03	80.44 ± 0.03	81.99 ± 0.03	79.33 ± 0.03	81.70 ± 0.03	77.02 ± 0.02	79.29 ± 0.03
w./o. Stratified alignment regularization	80.95 ± 0.03	84.21 ± 0.02	78.79 ± 0.03	81.44 ± 0.03	78.67 ± 0.03	80.41 ± 0.03	76.38 ± 0.03	78.30 ± 0.02
Ours	84.97 ± 0.03	87.09 ± 0.02	82.83 ± 0.03	84.92 ± 0.02	82.58 ± 0.03	85.34 ± 0.03	81.45 ± 0.03	83.35 ± 0.03

The PPMI Dataset provides an opportunity to assess robustness under multilingual and noisy speech conditions. Our model without Supervision and clinical alignment performs notably worse, achieving an mAP of 81.79 versus 84.97 from the full model. This suggests that dynamic context modeling is particularly effective at handling speech diversity and overlapping phonemes. A similar pattern is observed on UK Biobank Imaging Dataset, where removal of the same module reduces the F1 Score from 83.35 to 79.29, a 4.06-point degradation. This is attributed to UK Biobank Imaging Dataset's mix of prepared and spontaneous speech, which benefits from adaptive segment modeling. Notably, Stratified alignment regularization, responsible for progressive focal enhancement, also contributes significantly to overall robustness by emphasizing difficult regions during training. Across all datasets, its absence consistently leads to a reduction of more than 3 points in Recall and F1 Score, which reflects a loss in the model's ability to retrieve challenging or ambiguous patterns from the spectrogram representation. These findings validate the synergy of all three components in our design, each playing an indispensable role in elevating precision, recall, and generalization across speech modalities.

Beyond numerical performance, the ablation study also provides evidence for architectural balance and training stability. We observed that models without certain components tend to have higher standard deviation across runs, indicating reduced convergence stability. This is especially evident in the UK Biobank Imaging Dataset setup, where the removal of Supervision and clinical alignment leads to more variance in the Recall metric. Inference logs show that models with all components intact have fewer false positives on overlapping speech instances and improved consistency across speaker accents. These trends are in alignment with the advantages described in our method formulation, including the spectral anchoring strategy and segment-aware pooling. Crucially, our full model avoids the over-complexity seen in prior works, achieving superior performance with an optimized modular architecture rather than brute-force stacking. This supports our hypothesis that strategic integration of attention and adaptive mechanisms is more effective than merely increasing depth or parameter count. Thus, the ablation study not only confirms the individual necessity of each module but also highlights their collective importance in building a scalable, robust, and high-performing audio detection system.

The results presented in Table 8 demonstrate the robustness and reproducibility of our proposed model under different random initializations. We conducted five independent runs using fixed random seeds (10, 20, 30, 40, 50), each resulting in a complete training and evaluation cycle. Across these runs, we observed consistent outcomes in all key evaluation metrics, including accuracy, AUC, and F1-score. The standard deviation across runs remains low (like 0.62 for accuracy and 0.008 for AUC), and the computed variance values are minimal, indicating that the model's performance is not significantly influenced by the randomness in weight initialization or data shuffling. This level of consistency is especially important in the context of medical applications, where model stability is critical for clinical adoption. By explicitly reporting these metrics and repeating experiments across multiple seeds, we align with best practices in the field of reproducible deep learning research. Moreover, the low variance confirms that our model generalizes reliably and is not overly reliant on a favorable single run, which enhances the credibility of our experimental conclusions. This robustness further supports the effectiveness of our architectural design, optimization strategy, and data preprocessing pipeline.

**Table 8:** Performance across different random seeds for the proposed method.

**Metric**	**Mean**	**Standard deviation**	**Variance**
Accuracy (%)	91.3	0.62	0.38
AUC	0.947	0.008	0.000064
F1-score	0.902	0.012	0.000144

Each result is averaged over 5 runs with seeds 10, 20, 30, 40, and 50.

As shown in Table 9, our model demonstrates consistently strong performance when evaluated on patients in prodromal or early-stage disease categories, such as those diagnosed with mild cognitive impairment (MCI) or exhibiting a Clinical Dementia Rating (CDR) of 0.5–1.0. The model achieves AUC values exceeding 0.90 on both the ADNI-MCI and OASIS early-stage subgroups, with corresponding accuracy and F1-scores also remaining high. These results suggest that the proposed method is capable of capturing subtle neurodegenerative patterns that are often missed by conventional diagnostic approaches at this stage. The ability to detect early-stage pathological signals is particularly important for clinical translation, as it aligns with the goals of proactive intervention and disease-modifying treatment strategies. Early diagnosis offers a critical window for initiating lifestyle modifications, pharmacological interventions, and inclusion in clinical trials, where therapeutic impact is typically greater. The subgroup analysis further highlights the generalizability of our method across datasets, since performance remains stable despite differences in imaging protocols and population demographics. Importantly, this analysis supports the broader claim that our model is not only effective in distinguishing advanced disease stages but also in identifying early signs of cognitive decline. This enhances its potential role in real-world screening scenarios, especially in aging populations or high-risk groups where early detection is essential for reducing disease burden.

**Table 9:** Model performance on early-stage disease subgroup (MCI or CDR ≤ 1).

**Metric**	**Accuracy (%)**	**AUC**	**F1-score**
ADNI-MCI	89.4 ± 0.8	0.926 ± 0.009	0.883 ± 0.010
OASIS-CDR 0.5–1.0	87.1 ± 1.0	0.904 ± 0.011	0.864 ± 0.012

Table 10 summarizes the top-ranked radiomic features based on SHAP values, along with their corresponding anatomical regions. Notably, the features with the highest impact on model decisions are localized in the hippocampus, entorhinal cortex, and posterior cingulate–regions consistently implicated in the earliest stages of Alzheimer's disease pathology according to both imaging and histopathological studies. These areas are known to exhibit early atrophy, metabolic changes, and functional disconnection, making them critical targets for early diagnosis and monitoring. The prominence of entropy and texture-related features suggests that the model is sensitive to subtle structural and microarchitectural alterations that may precede gross anatomical shrinkage, which is particularly valuable for detecting prodromal cases. The alignment between model-driven feature importance and established neuropathological markers supports the interpretability and biological plausibility of our approach. This enhances confidence in the clinical relevance of the predictions, especially in screening scenarios where diagnostic transparency is critical.

**Table 10:** Top contributing radiomic features identified via SHAP values.

**Feature name**	**Anatomical region**	**SHAP importance**
GLCM entropy	Hippocampus	0.128
First-order skewness	Entorhinal cortex	0.103
GLRLM run length non-uniformity	Posterior cingulate	0.094
Wavelet energy (LLH)	Temporal lobe	0.087
First-order kurtosis	Parahippocampal Gyrus	0.079

As shown in Table 11, the proposed model maintains strong diagnostic performance on two external cohorts—UK Biobank and OASIS—demonstrating robust generalization to previously unseen populations and imaging protocols. Despite the inherent domain shift across these datasets, which differ significantly in scanner types, demographic distributions, and acquisition pipelines, the model achieved AUC values above 0.88 and maintained high classification accuracy and F1-scores. These results indicate that the learned representations are not overfitted to any specific dataset characteristics but instead reflect disease-relevant patterns that are consistent across domains. This level of performance under cross-dataset conditions suggests that the model is resilient to real-world variability, an essential property for clinical deployment. Moreover, the external testing setup—where no fine-tuning was performed on the target datasets—simulates real-world deployment scenarios in which labeled data may not be readily available for calibration. The ability to transfer effectively under such constraints further supports the translational potential of the proposed framework, especially for early-stage disease screening or integration into multi-center clinical workflows.

**Table 11:** External validation performance on UK Biobank and OASIS datasets.

**Dataset**	**Accuracy (%)**	**AUC**	**F1-score**
UK Biobank	84.3 ± 0.8	0.889 ± 0.009	0.864 ± 0.011
OASIS	86.7 ± 0.7	0.902 ± 0.008	0.879 ± 0.010

The model was trained on ADNI and PPMI only.

## 5 Conclusions and future work

In this study, we tackled the pressing challenge of early detection in neurodegenerative diseases, where subtle and heterogeneous brain deterioration patterns often evade conventional diagnostic models. To address this, we developed a comprehensive approach that integrates object detection with advanced radiomic analysis and disentangled representation learning. Our method is rooted in a hybrid representation framework that effectively decouples age-related changes from disease-specific alterations, thereby enhancing the specificity of extracted features. At the core of our solution lies NeuroFact-Net, a novel dual-path variational encoder-decoder architecture supervised along both anatomical and diagnostic dimensions. This model not only boosts interpretability but also enables dynamic tracking of disease progression through latent factor disentanglement. Complementing this, we introduced the Causal Disease-Aware Alignment (CDAA) mechanism, which ensures robust generalization across populations by enforcing contrastive, adversarial, and distributional consistency constraints. Validated on diverse multi-site MRI and PET datasets, our approach achieved diagnostic accuracy, transferability across imaging sites, and interpretability, showcasing strong potential for integration into clinical neuroimaging workflows.

Despite these promising outcomes, there are two notable limitations that warrant future exploration. While our method achieves disentanglement between age and disease-related features, the reliance on supervised signals for factor separation may introduce bias in populations with overlapping pathology and aging effects. Future work could explore self-supervised or semi-supervised extensions to reduce supervision dependency. Although CDAA enhances population-level alignment, it may underperform when faced with rare disease variants or imaging artifacts not well-represented in training data. Incorporating meta-learning or continual learning techniques could further strengthen robustness to such edge cases. Our study lays a foundation for causally-aware, interpretable AI systems in neuroimaging, with clear pathways for refinement and real-world deployment.

## Data Availability

The original contributions presented in the study are included in the article/supplementary material, further inquiries can be directed to the corresponding author.
